# Transforming the Niche: The Emerging Role of Extracellular Vesicles in Acute Myeloid Leukaemia Progression

**DOI:** 10.3390/ijms25084430

**Published:** 2024-04-17

**Authors:** Manuel Mendes, Ana C. Monteiro, Estrela Neto, Cristina C. Barrias, Manuel A. Sobrinho-Simões, Delfim Duarte, Hugo R. Caires

**Affiliations:** 1 i3S—Instituto de Investigação e Inovação em Saúde, Universidade do Porto, 4200-135 Porto, Portugal; manuel.mendes@i3s.up.pt (M.M.); ana.monteiro@i3s.up.pt (A.C.M.); estrela.neto@i3s.up.pt (E.N.); ccbarrias@i3s.up.pt (C.C.B.); massimoes@med.up.pt (M.A.S.-S.); delfimd@med.up.pt (D.D.); 2 ICBAS—Instituto de Ciências Biomédicas de Abel Salazar, Universidade do Porto, 4050-313 Porto, Portugal; 3 INEB—Instituto de Engenharia Biomédica, Universidade do Porto, 4200-135 Porto, Portugal; 4 IPATIMUP—Instituto de Patologia e Imunologia Molecular, Universidade do Porto, 4200-135 Porto, Portugal; 5 Department of Clinical Haematology, Centro Hospitalar Universitário de São João, 4200-319 Porto, Portugal; 6 Clinical Haematology, Department of Medicine, Faculdade de Medicina da Universidade do Porto (FMUP), 4200-319 Porto, Portugal; 7 Unit of Biochemistry, Department of Biomedicine, Faculdade de Medicina da Universidade do Porto (FMUP), 4200-319 Porto, Portugal; 8 Department of Hematology and Bone Marrow Transplantation, Instituto Português de Oncologia (IPO)-Porto, 4200-072 Porto, Portugal

**Keywords:** extracellular vesicles, exosomes, bone marrow microenvironment, acute myeloid leukaemia, organ-on-a-chip

## Abstract

Acute myeloid leukaemia (AML) management remains a significant challenge in oncology due to its low survival rates and high post-treatment relapse rates, mainly attributed to treatment-resistant leukaemic stem cells (LSCs) residing in bone marrow (BM) niches. This review offers an in-depth analysis of AML progression, highlighting the pivotal role of extracellular vesicles (EVs) in the dynamic remodelling of BM niche intercellular communication. We explore recent advancements elucidating the mechanisms through which EVs facilitate complex crosstalk, effectively promoting AML hallmarks and drug resistance. Adopting a temporal view, we chart the evolving landscape of EV-mediated interactions within the AML niche, underscoring the transformative potential of these insights for therapeutic intervention. Furthermore, the review discusses the emerging understanding of endothelial cell subsets’ impact across BM niches in shaping AML disease progression, adding another layer of complexity to the disease progression and treatment resistance. We highlight the potential of cutting-edge methodologies, such as organ-on-chip (OoC) and single-EV analysis technologies, to provide unprecedented insights into AML–niche interactions in a human setting. Leveraging accumulated insights into AML EV signalling to reconfigure BM niches and pioneer novel approaches to decipher the EV signalling networks that fuel AML within the human context could revolutionise the development of niche-targeted therapy for leukaemia eradication.

## 1. Introduction

Acute myeloid leukaemia (AML) is an aggressive blood cancer arising from the uncontrolled growth of immature myeloid cells in the bone marrow (BM) [1]. This disease carries a high mortality rate, with a five-year survival rate of only 24% and a median survival of just 8.5 months [2]. This dismal prognosis is primarily attributed to the frequent recurrence of the disease after initial treatment. This relapse occurs because a subpopulation of leukaemic stem cells (LSCs), resistant to conventional therapies, manages to evade eradication and persist within the BM [3].

LSCs possess a remarkable ability to hijack their surrounding microenvironment—the BM niche—to their advantage. This remodelling process seems to be orchestrated in part through extracellular vesicle (EV)-mediated communication, allowing LSCs to (i) ensure their autonomous survival within the BM environment; (ii) displace healthy haematopoietic stem cells (HSCs) from their niches, hindering the production of normal blood cells; and (iii) dramatically alter haematopoietic niches in support of uncontrolled progression. Importantly, this niche hijack sets the basis for LSCs to evade chemotherapy, lying dormant and undetected within the niche, only to re-emerge as a full-blown relapse later on.

## 2. Extracellular Vesicles

**Figure 1:**
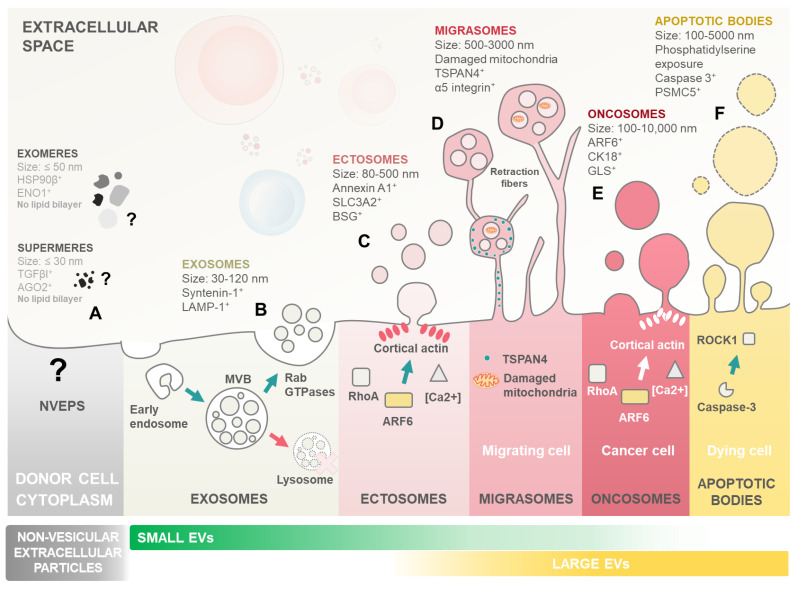
**Landscape of extracellular particle (EP) and extracellular vesicle (EV) subtypes.** Biogenesis and key characteristics of various EPs including both non-vesicular extracellular particles and a plethora of distinct EV subtypes. EVs are membrane-enclosed structures shed by all cell types, with critical roles in cell–cell communication and cargo delivery. Notably, leukaemic blasts can exploit EVs to reprogram marrow niche cells, promoting their own survival, proliferation, dormancy, and ultimately contributing to therapy resistance and relapse. (**A**) Non-vesicular extracellular particles (NVEPs) encompass a heterogeneous group of nanoparticles distinct from EVs. Unlike EVs, they lack a lipid bilayer membrane and are formed through various non-vesicular pathways. Examples include exomeres, shed from the plasma membrane, and supermeres, formed by protein aggregation. Symbol: (?) denotes the ill-defined nature of non-vesicular extracellular particles with largely unknown biogenesis and mechanisms of action. (**B**) Exosomes are the smallest EV subtype, ranging from 30 to 120 nm in diameter. They originate from the endosomal system. Invaginations of the limiting membrane of multivesicular bodies (MVBs) create intraluminal vesicles (ILVs) that become exosomes upon MVB fusion with the plasma membrane. Rab GTPases play a crucial role in directing MVB trafficking and exosome secretion, along with other essential molecules like SNARE proteins. (**C**) Ectosome/microvesicles are larger than exosomes, with a size range of 80–500 nm. Ectosomes bud directly outward from the plasma membrane in a Rho A- and ARF6-dependent process. Increased calcium concentration and cortical actin assembly at the budding site facilitate their formation. (**D**) Migrasomes are large EVs, ranging from 500 to 3000 nm. Migrasomes contain smaller EVs within their lumen and originate from the fragmentation of retraction fibres formed during cell migration. TSPAN4 proteins are crucial for migrasome formation, and damaged mitochondria are often found within them. (**E**) Oncosomes are a specialized type of ectosome released by cancer cells. Their biogenesis is highly heterogeneous and cancer-type-dependent, reflecting the diverse mechanisms employed by different cancers to manipulate their environment. They contribute to multiple hallmarks of cancer progression. (**F**) Apoptotic bodies, remnants of programmed cell death (apoptosis), are the largest EVs, ranging from 100 to 5000 nm. These vesicles form from the fragmentation of the apoptotic cell and are subsequently released. Caspase-3 and ROCK1 are key players in the apoptotic process that leads to the formation and release of apoptotic bodies.

Extracellular vesicles (EVs) are particles released from cells, delimited by a lipid bilayer, that cannot replicate on their own [4]. Importantly, EVs have been proposed to be fundamental in cell–cell communication in both healthy and pathological conditions [5,6,7]. There exist multiple types of EVs, which differ in their biogenesis, size, function, and the markers they express [8] (Figure 1). 

Although often discussed alongside non-vesicular extracellular particles (NVEPs), in the literature, EVs and NVEPs are fundamentally different. Unlike EVs, which are enclosed in lipid membranes, NVEPs lack this membrane structure, leading to very distinct biogenesis pathways. Consequently, the mechanisms underlying NVEP formation and function remain less understood compared to those of EVs [4,9] (Figure 1A). This review will focus on general EVs, considering the recent update performed on the minimal information for studies of extracellular vesicles (MISEV) guidelines from the International Society for Extracellular Vesicles [4].

Exosomes are among the most extensively studied subtypes of EVs (Figure 1B). Their biogenesis initiates with the formation of early endosomes integrating endocytic vesicles from plasma membrane invagination. These early endosomes, present within the cytoplasm, undergo a maturation process to become late endosomes or multivesicular bodies (MVBs). As part of this maturation, the endosome membrane invaginates to form intraluminal vesicles (ILVs) within the lumen of MVBs. These ILVs encapsulate various cytosolic components, including proteins and RNA. The sorting of cargo into them during the transition from early to late endosomes can occur through mechanisms both dependent on and independent of the Endosomal Sorting Complex Required for Transport (ESCRT) [10]. When MVBs fuse with the cellular membrane and release their content into the extracellular space, the released vesicles become what is referred to as exosomes [5,10].

Ectosomes (also known as microvesicles) are formed directly from the budding of the cellular membrane (Figure 1C). Ectosome formation is linked to cytoskeleton remodelling mechanisms, most notably the ARF6 GTP/GDP cycle [11]. A recent development in EV research was the identification of migrasomes (also known as pomegranate-like structures) (Figure 1D). Migrasomes are EVs composed of a large vesicle, containing smaller vesicles, that form during cell migration as retraction fibres are left behind by the cells. Migrasomes grow on the tips of retraction fibres and are released once these break [12]. Tetraspanin 4 (TSPAN4) was demonstrated to be essential in migrasome formation, is enriched in retraction fibres, and is currently considered the primary marker for these EVs [13].

Oncosomes are large EVs specifically secreted by cancer cells, distinguished by their size, typically ranging from 1 to 10 μm in diameter (Figure 1E). These vesicles carry a diverse cargo, including proteins, nucleic acids, and lipids, which they transport to neighbouring or distant cells, empowering the emergence of cancer hallmarks. Interestingly, Muralidharan-Chari et al. [14] showed the ARF6 GTP/GDP cycle to have a regulatory effect on ectosome and oncosome release in tumour cells. On the other hand, apoptotic bodies (Abs) are another class of EVs produced by apoptotic cells, from the fragmentation of the cell membrane, which contain their cellular components, including nucleic acids (Figure 1F). Abs have been shown to be involved in cellular communication and in DNA and RNA degradation [15]. To perform their cellular communication functions, EVs either interact directly with receptors at the membrane surface or are uptaken by their target cell [4,16]. EV uptake is dependent on conditions such as pH and temperature, and distinct methods for EV uptake have been proposed, such as endocytosis, phagocytosis, pinocytosis, and membrane fusion [17,18].

Importantly, cell secretion of these multiple EV subtypes is frequently altered in cancer and in leukaemia in particular. Indeed, illustrating the importance of deregulated EV signalling networks in AML, Szczepanski et al. showed that EV fractions isolated from the peripheral blood of AML patients exhibit a 60-fold increase in vesicular protein levels compared to healthy controls [19]. Indeed, in recent decades, leukaemic cell-derived EVs have been shown to contribute towards the progression of the AML, empowering virtually all known cancer hallmarks in the process [20,21,22,23,24,25,26,27].

Next, we will briefly describe the process of LSC transformation and highlight how the interaction between AML blasts and the BM niches through EV signalling is instrumental for disease progression but also for establishing reservoirs of residual disease and relapse upon therapy.

## 3. The Leukaemia Heist: Niche Remodelling Favours Disease Progression

### 3.1. Leukaemic Stem Cell Inception

**Figure 2:**
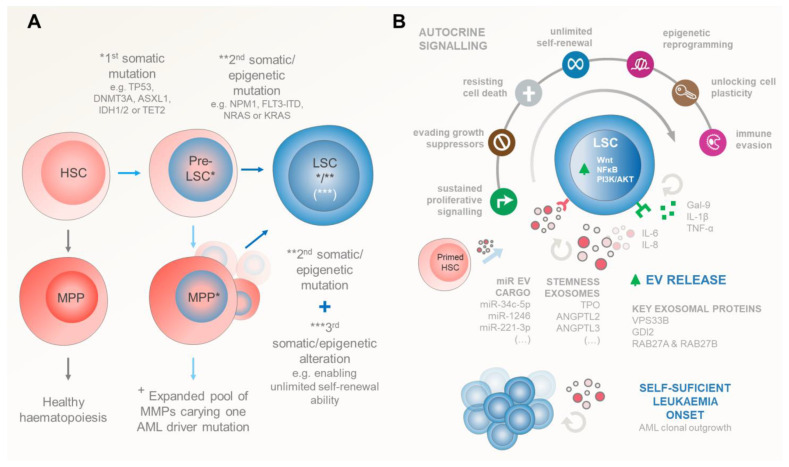
**Genetic and/or epigenetic events driving the inception of AML.** (**A**) Stepwise progression from healthy HSCs to LSCs. Initially, HSCs can accumulate the first genetic or epigenetic “hit”, leading to the formation of pre-leukaemic stem cells (pre-LSCs). These pre-LSCs expand within the BM environment, carrying mutations in tumour suppressor genes (e.g., TP53) and epigenetic regulators (e.g., DNMT3A, ASXL1, IDH1/2, and TET2). Additional mutations (the “second hit”) occur in pre-LSCs, affecting genes that control HSC quiescence, proliferation, self-renewal, differentiation, or epigenetic regulation. Accumulation of these two sets of mutations results in the emergence of full-blown AML. LSCs can also arise from pre-leukaemic multipotent progenitors (MPPs) that acquire a second genetic or epigenetic alteration concomitantly with a third event to regain HSCs’ self-renewal ability. (**B**) Upon these genetic events, the successful establishment of LSCs relies on their autocrine signalling ability to survive. This early survival is reliant mostly on autocrine EV signalling (alongside with HSCs and pre-LSC counterparts) that shed exosomes enriched in stemness factors such as TPO, ANGPTL2/3, and other EVs carrying pro-leukaemic miRs that upregulate LSCs’ Wnt, NFkB, and/or PI3K/AKT pathways, driving unchecked AML blast cell proliferation. EV shedding is exacerbated in transformed LSCs compared to healthy HSC counterparts, highlighting the importance of this mechanism for the onset of AML. Autocrine EV endorsement of multiple leukaemic cancer hallmarks is further complemented by autocrine secretion of several soluble cytokines. Arguably, many of those cytokines can also be caried in the EV luminal compartment. Symbols: (*) represents the “first hit” early mutations occurring in HSCs/MPPs that give rise to pre-LSCs; (**) illustrates the “second hit” mutations that pre-LSCs accumulate in order to transform in LSCs; in the case of pre-transformed MPPs (***) denotes the existence of a “third event” in genes that enables MPPs to step back and re-gain the unlimited self-reviewal ability of HSCs. (+) Somatic alterations in HSCs give rise to an expanded pool of MPPs carrying at least one AML driver mutation facilitating the acquisition of additional alterations required for AML establishment.

Aging and/or multiple stress factors predispose niche-located HSCs and progenitor cells to stochastically accumulate somatic DNA mutations. Importantly, in AML, the LSCs arise when these mutations are acquired—in a stepwise manner—in genes that control HSCs’ (i) quiescence and proliferation (FLT3, RAS, P53, c-KIT, and STAT3), (ii) self-renewal and differentiation (NPM1, RUNX1, CEBPA and other myeloid transcription factors), and (iii) epigenetic regulation (DNMT3A, DNMT3B, DNMT1, TET1, TET2, IDH1, IDH2) [28,29,30] (Figure 2A).

Notably, these gene alterations are gained due to either chromosomal rearrangements (55%) or as point mutations in cytogenetically normal (45%) patients. A seminal work by Papaemmanuil et al. identified 5234 driver mutations across 76 genes/genomic regions, with 2 or more drivers being present in 86% of the 1540 analysed patients [29]. Interestingly, despite the notably heterogenous AML genetic profile, the Cancer Genome Atlas Research Network found that the overall mutational burden in AML is relatively low compared to other malignancies. Indeed, this ground-breaking work determined that leukaemic blasts bear, on average, 13 gene mutations, 5 of which are in genes that are recurrently mutated in AML [28]. These include mutations in NPM1, FLT3, DNMT3A, IDH1/2, and NRAS/KRAS driver genes, highlighting their importance for initiating leukaemogenesis.

Consistently, data from myelodysplastic syndrome (MDS) and AML patient cohorts identified a number of tumour suppressor (TP53) and epigenetic mutations (DNMT3A, ASXL1, IDH1/2, and TET2) as early (first hit) initiating events preceding leukaemic transformation. This early event provides a selective advantage for the pre-leukaemic HSC clonal expansion within the BM environment [31,32,33,34]. Later on, full-blown AML emerges upon accumulation of additional mutations (second hit) in NPM1 or in the FLT3 and NRAS signalling pathways in the pool of expanded pre-leukaemic HSCs. Interestingly, AML can also arise from pre-leukaemic multipotent progenitors (MPPs) and/or committed myeloid/lymphoid progenitor cells (CMPs, GMPs, and CLPs), where additional genetic/epigenetic alterations are required to regain HSCs’ self-renewal ability. Interestingly, in this scenario, leukaemic progenitor cells (LPCs) assemble a complex and self-sufficient hierarchy whose signature has prognostic value [35,36].

Indeed, this seems to support the prevailing hypothesis suggesting that AML does not originate from a solitary LSC but rather from multiple subclones organized in linear or branched structures, each exhibiting distinct mutations and/or epigenetic alterations [37,38]. Interestingly, this view implies that each of these leukaemic clones is sustained by their own reservoir of LSCs operating in their niches. Although altered genotype is of capital importance for AML onset, its progression and clinical manifestation is determined by the complex microenvironment within the haematopoietic BM niches [39]. Next, we will address how initiating leukaemia maintains LSCs and their progeny, educates non-haematopoietic cells, and remodels the BM niches to sustain disease progression while outcompeting HSCs’ niche residency and suppressing haematopoiesis.

### 3.2. Self-Sustained Leukaemia Growth

Despite partial dependence on signals from the haematopoiesis-regulating BM microenvironment for survival and proliferation, LSCs can thrive on their own, outcompete HSCs, and occupy their niches (Figure 2B). Indeed, several autocrine stimulatory mechanisms have been identified in promoting LSC self-sufficiency, including Gal-9, TNF-α, IL1-β, IL-6, IL-8, NmU peptides, and EVs (Table 1). Very recently, the Theresa Whiteside group [40] showed that small EVs in the plasma of cancer patients and healthy donors had 51 detectable cytokines/chemokines/soluble receptors/growth factors, with 40 of those being carried as EV luminal proteins. Most interestingly, these included TNF-receptor II, IL-6/IL-6 receptor, IL-8, and SDF-1α. Arguably, this may imply that EV signalling also plays a critical role in many of the leukaemia-promoting pathways that were originally thought to be cytokine-exclusive.

Upon transformation, LSCs actively secrete Gal-9 to bind TIM-3 receptors in their membrane, providing a constitutive autocrine stimulatory loop to activate NF-κB and canonical Wnt pathways and driving the self-renewal of LSCs [41,42]. The constitutive NF-κB activity in these initiating leukaemic cells seems to be further maintained through autocrine TNF-α and IL1-β secretion [43,44,45]. Interestingly, IL1-β autocrine feedback also triggers AML blasts to secrete HGFs, GM-CSF, IL-6, and TNF-α, further reinforcing the uncontrolled proliferative signalling network in AML. This seems to be complemented by the autocrine secretion of Neuromedin U (NmU) for downstream MYB transcription in LSCs [46]. Importantly, MYB favours cell proliferation and activates cellular oncogenic programs that drive the well-known overexpression of BCL2, MYC, GFI1, MTL5, and IKZF1 in leukaemic blasts [46,47,48].

**Table 1:** Factors influencing leukaemia progression: origins, targets, and reprogramed niche signalling pathways. Leukaemia self-sufficiency (blue background); Endosteal niche (brown background); Vascular niche (light red background); Unknown EV-mechanism of action (grey background).

Factor	Origin	Target	Signalling	Pro-Leukaemia Effect	Cancer Hallmark [20]	Ref.	Sample Origin
LEUKAEMIA SELF-SUFFICIENCY							
Gal-9	LSCs	LSCs	Autocrine	Promote LSC self-renewal through activation of NF-kB and Wnt pathways, by Gal-9/TIM-3 binding.	Enabling replicative immortality	[41]	Patient Sample
TNF-α	LSCs	LSCs	Autocrine	Promote LSC self-renewal through increase in NF-kB pathway activity in positive feedback loop.	Enabling replicative immortality	[43]	Mouse BM
IL1-β	LSCs	LSCs	Autocrine	Promote LSC self-renewal through increase in NF-kB pathway activity and increase in HGFs, GM-CSF, IL-6, and TNF-α production, creating a positive feedback loop of proliferative signalling.	Enabling replicative immortality	[45]	Patient Sample
NmU	LSC	LSC	Autocrine	Promote leukaemic cell growth and proliferation through MYB-related mechanism.	Sustaining proliferative signalling	[46]	K562 AML Line, Patient Sample
miR-221- 3p	AML Cells	AML Cells	Autocrine EV Cargo	Promote leukaemic cell growth and proliferation through promoting entry into cell cycle and apoptosis inhibition through downregulation of Gpb2 gene expression.	Resisting cell death	[49]	Mouse Model THP-1, HL-60, Kasumi-1, MOLM-13 (AML Cell Lines)
ENDOSTEAL NICHE							
IL-8	HSCs, BMMSCs	LSCs	Soluble Factor	Lead to induction of proliferative and oncogenic pathways and recruitment of myeloid-derived suppressor cells through binding to overexpressed CXCR1 and CXCR2 receptors.	Sustaining proliferative signalling	[50]	Patient Sample
MIF	AML Blasts	BMMSCs	Soluble Factor	Induce IL-8 secretion in PKCβ-regulated mechanism.	Sustaining proliferative signalling	[51]	Patient Sample
IL-6	BMMSCs	AML cells	Soluble Factor	Promote chemoresistance in leukaemic cells through STAT-3 pathway activation, leading to higher OXPHOS levels.	Resisting cell death	[52]	HS-5 (BMMSC lines) HL-60, U-937,THP-1 (AML lines)
TPO	Niche Osteoblasts, HSCs, LSCs	HSCs and LSCs	EV Cargo	Induce HSC adhesion to osteoblastic niche. Promote SC quiescence and induce SC proliferation in endosteal niche.	Enabling replicative immortality	[53,54]	Mouse BM
ANGPTL3	Endothelial cells, BMMSCs, HSCs, LSCs	HSCs and LSCs	EV Cargo	Directly bind to HSCs. Promote SC quiescence through suppression of TF Ikaros.	Enabling replicative immortality	[53,55]	Mouse BM
miR-34c-5p	LSCs	None, exported out of LSCs via EVs	EV Cargo	miR-34c-5p induces LSC senescence through p53/p21-dependent CDK/Cyclin or p53-independent CDK/Cyclin pathways. LSC EV-mediated export of this factor inhibits this effect, leading to worse AML prognosis.	Senescent cells evading growth suppressors	[56]	Patient Sample
miR-1246	AML Cells	LSCs	EV Cargo	Activate STAT3 pathway through LRIGH1 downregulation. Increase LSC viability and proliferation. Decrease LSC differentiation and apoptosis.	Resisting cell death	[57]	KG1-A, Kasumi-1 (AML lines)
IFN-γ	BMMSCs, AML cells	BMMSCs, HSCs and LSCs	Soluble factor EV Cargo	Pro-inflammatory effects. Activate STAT1 signalling to induce oxidative stress, by increased ROS production, leading to decreased osteogenic differentiation of MSCs. Decrease immune response to LSCs by conditioning MSCs into anti-inflammatory activity as a response to excess IFN-γ.	Tumour promoting inflammation Avoiding immune destruction	[58,59]	Patient Sample
PGE2 TGF-β TSG-6 HGF HLA-G6 IL-10 IL-6 galectins	BMMSCs	Many	Soluble factor EV Cargo	Dampen immune response against LSCs through promoting anti-inflammatory environment as response to excess inflammatory factors produced by AML cells.	Avoiding immune destruction	[60,61,62]	Patient Sample [60,62] Mouse BM [61] Mouse MS-5 Stromal Line [62]
miR-188-5p	LSCs	BMMSCs	EV Cargo	Promote LSC proliferation through restructuring of niche MSCs, as they increase MCAM presence on their surface, increasing binding to myeloid cells, leading to ERK signalling pathway activation.	Sustaining proliferative signalling	[63]	KG1a, SKM-1 (AML Lines) HS5, HS27a (BMMSC lines)
miR-4532	AML Blasts	Pre-Osteoblasts	EV Cargo	Increase DKK1 expression, which inhibits Wnt pathway signalling, leading to decrease in osteoblastic differentiation, causing disruption of endosteal niche bone formation and normal haematopoiesis.	Activating invasion	[64,65]	HL-60, Molm-14, OCI-AML3 (AML lines)
PRDX2 PRDX4 L-plastin	Erythroleukaemia Cells	Osteoclast precursors	EV Cargo	Promote bone resorption through induction of osteoclast differentiation. Bone resorption increases the central marrow cavity space, where AML cell growth can occur.	Activating invasion	[66,67,68]	Mouse BM Human Breast Cancer lines
YBX1	AML Cells	BMMSCs	EV Cargo	Reduces osteoblastic differentiation, disrupting normal haematopoiesis. YBX1 downregulation led to impact on other EV cargo, hinting at possible cooperation between different factors.	Activating invasion	[69]	K562, MV-4–11 (AML Lines) Patient Sample (BMMSC)
FTO	BMMSCs	AML Blasts	EV Cargo	Increased LncRNA GLCC1 expression in AML blasts, leading to increase in LncRNA-GLCC1-IGF2BP1-c-Myc signalling pathway activation, linked with higher tumour aggressiveness.	Sustaining proliferative signalling	[70]	THP-1, Kasumi-1 (AML Lines) Patient Sample (BMMSC)
AML derived EVs	AML Cells	BMMSCs	EVs	Alter gene expression profile of BMMSCs, with concentration-dependent effects. Increased MSC survival, proliferation, and metabolic activity through increased Ki-67 and BCL2 expression (at lower concentrations of AML cell-derived EVs). Downregulation of ROS production. Upregulation of apoptosis (at higher AML cell-derived EV concentrations).	Resisting cell death	[71]	Patient Sample
VASCULAR NICHE							
CXCL12 (SDF-1)	BMMSCs	AML Cells	Soluble Factor EV Cargo	Bind to CXCR4 expressed on AML cells to promote homing to BM niche and stromal cell–AML cell adhesion. Increase AML cell resistance to apoptosis. Promote LSC quiescence, maintenance, and proliferation.	Activating invasion Resisting cell death	[72,73,74]	Patient Sample (BMMSCs) KG-1a (AML Line) [72] Mouse BM [74]
ANGPL2	Endothelial Cells	LSCs	EV Cargo	Bind to LILRB2 receptor to promote LSC maintenance and drive LSCs to localize around endothelial cells in BM niche.	Enabling replicative immortality	[75]	Mouse Model
VEGF VEGFR	AML Cells	Endothelial Cells	EV Cargo	Promote vascular remodelling and angiogenesis.	Inducing or accessing vasculature	[25]	Patient Sample HUVECs
IGF-1R coding mRNA	AML Cells	BMMSCs	EV Cargo	Promote IGF-1R expression, which increases VEGF secretion, leading to increased angiogenesis and proliferation.	Inducing or accessing vasculature	[76]	HEL, HL-60, Molm-14, U937 (AML Lines) Patient Samples
miR-92a	AML Cells	Endothelial Cells	EV Cargo	Promote endothelial cell migration and tube formation, but not growth. Decrease expression of the pro-angiogenic Integrin-α5.	Inducing or accessing vasculature	[77]	K562 AML Line HUVECs
miR-3064-3p miR-339-5p miR-3622a-5p	AML Cells	Endothelial Cells	EV Cargo	Promote angiogenesis in HUVECs, regulated by P62 expression.	Inducing or accessing vasculature	[78]	U937 AML Line HUVECs
CXCL12 SCF IL-7 IL-15 M-CSF BMP-4 CCL-2	BM Adipocytes	BMMSCs	Soluble Factors	Promote HSC proliferation and haematopoietic regeneration, upregulated and hijacked in AML.	Sustaining proliferative signalling	[79]	Mouse Model
GDF15	AML Cells	BM Adipocytes	Soluble Factor	Induce lipolysis in BM adipocytes, releasing fatty acids (FAs) into the vascular environment. FAs are uptaken by AML blasts via an FABP4-dependent mechanism and used as an energy source.	Deregulating cellular metabolism	[80]	THP-1, K562, HEL, HL-60 and Kasumi AML Lines Patient Sample (BMMSCs) differentiated into Adipocytes
IL-8 CCL2 TIMP-1 TIMP-2 VEGF-D	ADSCs	Endothelial Cells	EV Cargo	Induce tube formation and angiogenesis.	Inducing or accessing vasculature	[81]	Canine adipose tissue sample SVEC-4 Mouse endothelial line
miR-155-5p miR-106a-5p miR-106b-5p miR130b-3p miR-16-5p miR-181a-5p miR-19b-3p miR-466k miR-93-5p miR-126a-5p	AML Cells	HPSCs	EV Cargo	Induce activation of inflammatory secretion profiles in HPSCs, leading to increased AML progression.	Avoiding immune destruction	[82]	C1498 Mouse AML Line Mouse Model
UNKNOWN MECHAMISM							
MPIF-1 (CCL23)	Unknown	Found in blood plasma	Soluble Factor	Found at elevated levels in AML patient plasma. MPIF has reported to inhibit proliferation and differentiation of myeloid progenitors, but role in AML has not been described.	Unknown	[83]	Patient Sample
BMP10 CCL3 CX3CL1 OPN CD105 PTHLH CHRDL1 MMP7	Many	Found in blood plasma	Soluble Factors	Found at elevated levels in AML patient plasma. Factors linked with bone homeostasis through multiple different pathways. Potential coordinated mechanism of action in BM niche activity in AML	Unknown	[83]	Patient Sample
CD31/endomucin-expressing cellular debris particles	Endothelium	Found in vascular lumen	EVs	Particles of endothelial EV origin found in the vasculature of leukaemic mice, but not in healthy control specimens, suggesting it is a possible risk factor.	Unknown	[84]	Mouse model

Additionally, in MDS and AML patients, aberrant haematopoietic stem cells (HSCs) and progenitors (HSCPs), along with non-haematopoietic niche cells, were primed to secrete high levels of IL-8 [50,85]. Interestingly, IL-8 binding to CXCR1/CXCR2 receptors activates oncogenic STAT3 and PI3K/AKT signalling in LSCs and enhances myeloid-derived suppressor cell recruitment [86]. IL-6 secreted by leukaemic cells also activates STAT3 signalling, further triggers the oxidative phosphorylation metabolic pathway [52], and promotes CD36 expression, CD36-mediated uptake of fatty acids, and AML blast chemoresistance against Ara-c [87]. Interestingly, increased plasmatic IL-6 and IL-8 levels are both independent risk factors for AML prognosis and correlate with adverse outcomes in these patients [88]. For a more thorough insight on the soluble cytokine network in AML, please refer to recent reviews elsewhere [89].

In early stages, LSC-derived EVs can play a critical role in maintaining their own stemness. Indeed, inspiring work from Gu et al. identified that TPO, ANGPTL2, and ANGPTL3 stemness-related secretory proteins are carried in exosomes for this purpose in both HSCs and LSC counterparts [53]. Importantly, using silencing in vivo experiments, these authors demonstrated that VPS33B/GDI2 proteins regulate exosome maturation and release these pivotal proteins to maintain HSC stemness in an autocrine manner. Most importantly, HSCs secrete much higher levels of TPO, ANGPTL2, and ANGPTL3 compared with endothelial cells (and possibly other niche cells), with VPS33B knockdown in the latter not affecting the HSC phenotype. This evidence further reinforces the notion of a predominant autocrine mechanism for stem cell maintenance [53,55,75,90].

This highlights the critical role of autocrine exosome secretion in controlling HSC stemness, a mechanism hijacked by leukaemia-initiating cells (LICs) to thrive in their early stages. Consistently, silencing exosome-assembly protein VPS33B in both human AML cell lines (HL-60, THP-1, and U937) and AML patient samples led to a significant delay in cell proliferation and increased apoptosis in both CD34+-enriched LICs and bulk leukaemic blasts [53]. Additionally, it drastically affected leukaemia repopulation in an MLL-AF9-transduced AML mouse model. Similarly, lentivirus-mediated knockdown of Rab27a—involved in exosome biogenesis—decreased EV levels and significantly prolonged AML mouse survival [91]. Most interestingly, LSCs seem to use exosome export machinery to diminish intracellular levels of tumour suppressor elements as a means to escape control. Consistently, Chen et al. showed that LSCs overexpress RAB27B, a gene regulating exosome secretion, which is associated with poor prognosis in AML patients [92]. Importantly, the authors demonstrate that increased RAB27B in LSCs prevents their senescence and maintains their stemness both in vitro and in vivo. Mechanistically, LSCs seem to selectively promote the loading and release of exosomes rich in senescence-inducing proteins and other elements, such as miR-34c-5p, bypassing p53/p21/cyclin-dependent or p53-independent tumour suppressor checkpoints [56,92]. Interestingly, AML cell-derived EVs carrying miR-1246 also seem to target both LSCs and other leukaemic blasts. Upon internalization, these EVs activate the STAT3 pathway by targeting LRIG1, thereby promoting LSCs’ viability and colony formation ability while inhibiting cell apoptosis and influencing differentiation, ultimately augmenting the survival of LSCs [57]. Along the same line, Li et al. have recently observed that AML cell-derived small EVs—which are highly enriched in miR-221-3p—target Gbp2 gene expression on other AML blasts to promote AML cell proliferation and leukaemogenesis by accelerating cell cycle entry and inhibiting apoptosis on their own [49].

Additionally, Fang et al. have recently analysed the dysregulated expression of immune-related and exosome-related gene profiles for 151 AML patients from a public TCGA database. The authors have described a prognostic signature based on the expression of CD37, NUCB2, LSP1, MGST1, and PLXNB1 exosome-related genes and patients’ clinical outcomes [93].

Taken together, the rise of LSCs occurs due to the accumulation of a set of genetic mutations and/or epigenetic alterations which endow the cells with replicative immortality, unconstrained proliferation, and permanent immaturity. Upon these events, LSCs assure their initial survival via aberrant Gal-9, TNF-α, IL1-β, NmU, and IL-8 expression and stemness–exosomal autocrine signalling, followed by an induction of BM niche microenvironment remodelling through an intricate EV signalling network. Indeed, accumulated evidence strongly suggests that VPS33B, GDI2, RAB27B, and other exosome-associated proteins may play a greater part in early-stage leukaemia development than in normal haematopoiesis. This is a critical step to favour the unchecked leukaemic overcrowd of the marrow space, preceding mobilization to the peripheral blood and secondary organs [94].

### 3.3. Rewiring the Haematopoietic Niches for LSC Traction

The haematopoietic niche refers to the cellular and molecular environment that maintains HSC self-renewal and multipotency in the adult BM. It can be divided between different types, based on their location: the endosteal niche, within the interior bone or on the endosteum surface, and the vascular/perivascular niche, mostly located in the medullary compartment of the BM. The native endosteal niche extracellular matrix (ECM) is mostly rigid, at 35–40 KPa, and comprises collagen (types I and IV), fibronectin, and osteopontin [95,96]. On the other hand, the vascular niche ECM, which surrounds all the different cells of this niche, is mostly compliant, at 0.1 to 0.3 KPa, comprising laminin, collagen type IV, and fibronectin, and is thus softer than the endosteal niche ECM [95,96]. Noteworthy, the extracellular matrix of the different haematopoietic niches—along with their mechanical and chemical properties—have been shown to instruct niche cell phenotypes by modulating their properties, secretome, and function [97].

The reliance of LSCs on EV signalling networks to hijack the marrow environment prior to overt AML has now been experimentally demonstrated in several mouse models [82,91,94]. Globally, infusion of AML cell-derived EVs alone is able to trigger the same pathognomonic BM alterations—abnormal accumulation of osteoprogenitors, reduced mature osteoblast numbers and bone formation, and loss of HSC support ability—as the leukaemic blast cells themselves, highlighting their key role in reprogramming the niche signalling network.

**Figure 3:**
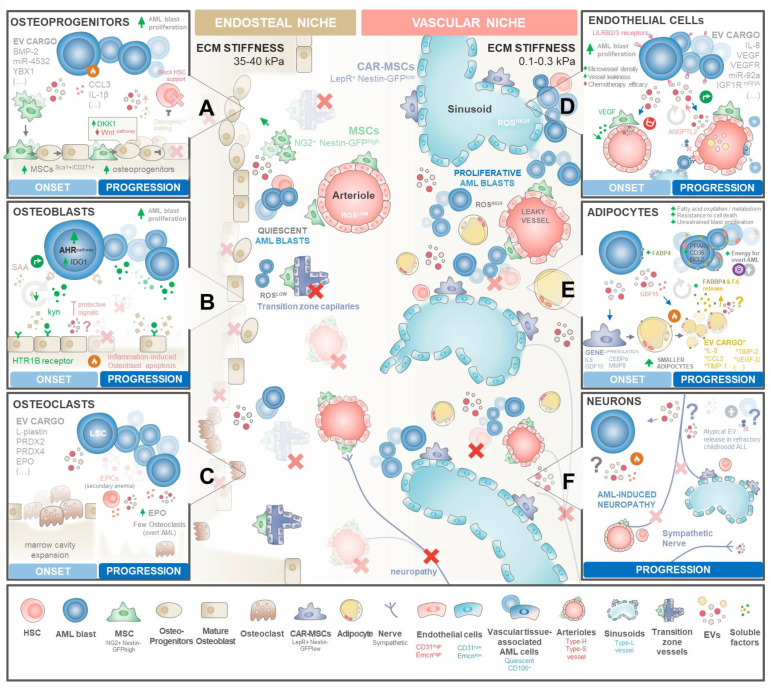
**Mapping EV signalling networks driving AML.** Blueprints of BM niche reprograming in distinct stages of BM infiltration. Healthy BM is organized in two functionally distinct compartments: a stiffer endosteal niche at the inner surface of the bone and the vascular niche located at the core of marrows’ cavity. Both haematopoietic niches are required for HSCs’ maintenance, proliferation, and maturation along lymphoid and myeloid lineages. AML onset severely remodels the BM microenvironment, causing (i) abnormal accumulation of osteoprogenitors and the (ii) destruction of the lining endosteum and endosteal vasculature. This leads to (iii) the expansion of central cavity space available for AML proliferation supported by an abnormal-vascularization BM core. Drastic remodelling of haematopoietic niches drives AML blasts to outcompete healthy HSCs and progenitors, compromising normal haematopoiesis. (**A**) AML blasts shed high amounts of EVs carrying pro-inflammatory and osteogenic factors, including BMP-2, miR-4532, or YBX1, that drive BMMSCs to expand and differentiate into osteoprogenitors. However, several of these EVs increase DKK1 expression at osteoprogenitors, decreasing the Wnt signalling required for the osteoblast maturation step. Osteolineage differentiation stalling causes abnormal accumulation of osteoprogenitors, which typically provide signalling molecules required for HSC maintenance, activation, and proliferation. (**B**) On the other hand, mature osteoblasts—that seem to be more involved in providing support for HSC maturation and differentiation—are typically destroyed by the inflammatory environment throughout disease progression. Early on, osteoblasts seem to be protective against AML. AML blasts balance this roadblock by exploiting the kynurenine(kyn)–HTR1B–SAA–IDO1 axis by secreting exacerbated amounts of kynurenine that interact with mature osteoblast receptor HTR1B, triggering the release of SAA that in turn activates the AHR pathway in AML blasts with transcription of IDO1 and other molecules that stimulate AML proliferation. With increasing clone expansion and inflammatory-driven cell death of lining osteoblasts over time, the kyn-SAA positive feedback loop eventually surpasses the inhibitory secretion profile of osteoblasts towards overt disease. (**C**) During AML establishment, LSCs seem to secrete EVs carrying L-plastin, PDRX2/4, and EPO that transiently increase the number of osteoclasts degrading the endosteal bone. This may assist the thriving of AML to overt disease by expanding the available marrow cavity space for clonal expansion. During disease progression, osteoclast numbers decrease, yet their activity may still be endorsed by high EPO levels secreted by expanded AML clones and by erythroid progenitor cells (EPCs) facing secondary anaemia stimuli triggered by haemopoiesis disruption by high AML infiltration. (**D**) LSCs secrete EVs carrying pro-angiogenic factors such as VEGF, VEGFR, IL-8, miR92-a, or IGF1R mRNA towards central niche vasculature. These EVs seem to target both perivascular MSCs and pericytes (that in return secrete high amounts of VEGF) and endothelial cells. The exacerbated pro-angiogenic microenvironment triggers an abnormal angiogenic process that drives the increased microvessel density with the formation of disorganized and leaky microcapillaries. In return, endothelial cells shed ANGPL2 + EVs that interact with LILRB2/4 receptors at AML blasts to reinforce their proliferation. The exposure of the remaining healthy HSCs to high reactive oxygen species (ROS) levels triggers their displacement from the niche with a possible exhaustion of the pool of quiescent HSCs. On the other hand, a residual reservoir of LSCs is guaranteed through the incorporation of AML blasts into the vasculature as quiescent tissue-associated AML cells. Their remarkable plasticity ensures a steady reservoir of residual disease. (**E**) Arguably, AML blasts shedding EVs seem to trigger certain MSC subsets to differentiate into adipocytes, losing their ability to support healthy HSCs. Subsequently, AML reprograms these adipocytes via GDF15, yielding smaller adipocytes that release fatty acids (FAs) into the vascular milieu. Upregulated FABP4 in nearby AML blasts transports these FAs to the mitochondria, generating the energy needed for overt leukaemia growth. In return, adipocytes secrete factors that endorse AML blast proliferation and resistance of cell death. (**F**) Sympathetic neurons are typically destroyed in the late stages of blast infiltration, originating generalised marrow neuropathy. The mechanisms of neuronal targeting in AML remains largely unexplored. Some reports observed in other haematological malignancies that neurons secrete atypical EVs in therapy-refractory patients in childhood acute lymphoblastic leukaemia (ALL). Symbols: (X) elements of the bone marrow niche that are frequently obliterated in AML; (?) denotes the largely unexplored nature of EV-mediated communication in AML disease.

Recent ground-breaking studies exploring single-cell RNA sequencing (scRNA-seq) and spatially resolved transcriptomics have facilitated a comprehensive exploration of the intricate landscape of heterogeneous stromal populations within BM [79], illustrating how they are reprogramed by AML blasts [83,98,99,100]. This has indeed been a hot topic in the quest to develop niche targeting therapies for LSC eradication. In this sense, Baryawno et al. elegantly conducted a comparative analysis of scRNA-seq profiles between healthy and MLL-AF9-knock-in leukaemic mice [98]. Their findings delineated 17 distinct BM populations at steady state, encompassing endothelial, pericyte, osteolineage, fibroblastic, mesenchymal, and chondrocyte clusters. Notably, the MLL-AF9 mouse model exhibited significant alterations in the cellular composition of the BM microenvironment, marked by a reduction in osteolineage-differentiated LepR-MSCs and a concurrent increase in osteoblast progenitors. While RNA-seq studies have provided valuable insights, Çelik et al. recognized the importance of proteome-based analysis to unveil molecules regulated by post-transcriptional mechanisms [83]. Utilizing the SOMAscan assay, they conducted proteomic profiling of the noncellular soluble compartment of the BM microenvironment in AML patients, revealing dysregulation of 91 upregulated and 77 downregulated proteins. The AML BM niche’s proteomic signature indicated perturbations in signalling networks, particularly those associated with chemokine and cytokine signalling, with notable alterations in proteins linked to bone homeostasis. This includes key regulators of the BMP signalling pathway, such as BMP10, CCL3, CX3CL1, osteopontin (OPN), endoglin (CD105), parathyroid hormone-like hormone, chordin-like protein 1, and matrix metalloproteinase 7 (MMP-7). While further research is required to elucidate BM stromal cell changes across distinct AML subtypes and stages, here, we will construct the blueprints of currently known AML signalling networks that reconfigure the endosteal and vascular niches which a special focus on EV-mediated intercellular communication (Figure 3).

#### 3.3.1. Endosteal Niche Remodelling

Many of the cytokines (including TNF-α, IL1-β, IFN-γ) and EVs, shed by LSCs for self-sustained growth concomitantly induces a pro-inflammatory and anti-angiogenic milieu in endosteal regions. This is an important landmark for re-educating endosteal niche mesenchymal stromal/stem cells, osteoprogenitors, endothelial cells, and, as recently shown, even healthy HSPCs [82] into a pro-leukaemic phenotype. This crosstalk triggers an extensive remodelling of the endosteum, yielding impaired osteoblastic differentiation with deficient bone mineralization while compromising the niche’s vasculature, suppressing its ability to maintain and properly regulate haematopoiesis. Next, we will address how the secretome and EVs shed by LSCs re-educate these surrounding cells and how leukaemia capitalizes on this crosstalk for disease progression.

##### BM Mesenchymal Stromal/Stem Cells

Bone marrow MSCs (BMMSCs) within the BM niches exhibit functional differences based on their location, exposure to vascular flow, oxygen conditions, and ECM stiffness [reviewed in [101,102]]. Consistently, several lines of evidence have shown that the EV secretion profile of BMMSCs is heavily influenced by inflammatory [60], oxygenation [103], and mechanical [104] microenvironmental features. Due to their plasticity, BMMSCs and their differentiated progeny represent one of the most critical components of the haematopoietic niches, comprising approximately 20% of the marrow’s cellular volume [105].

BMMSCs correspond to a heterogeneous population of cells that give rise to osteoblasts, chondrocytes, and adipocytes, with several subsets considered critical for the maintenance and homeostatic regulation of HSCs [106,107]. In this regard, two distinct subsets of MSCs have been identified based on their localization within the BM microenvironment: (i) Nestin-GFPhi NG2+ MSCs associated with endosteal transition zone vessels and arterioles, and (ii) Nestin-GFPlo LEPR+ [108,109] CXCL12-abundant reticular (CAR) [110,111] BMMSCs linked with sinusoids [105] in the central BM (Figure 3). These subsets provide hierarchical insights into the specialized niches within the BM and their roles in regulating both HSC and LSC functions. These stromal cells are major sources of stem cell factor (SCF) and interleukin-7 and are considered critical regulators of HSCs and several multipotent progenitors [99,111,112,113].

However, upon LSC onset, BMMSCs have been shown to undergo severe reprogramming through various interconnected mechanisms, including leukaemia-secreted factors, EV mediation, and cell-to-cell interactions [22,58,114,115]. Most interestingly, BMMSCs isolated from patients with AML—but not acute lymphoblastic leukaemia (ALL), Hodgkin disease (HD), or non-Hodgkin lymphoma—showed both transient and prolonged abnormal biological properties compared with healthy donor counterparts [116]. These included high heterogeneity in cell morphology [117], limited proliferation capacity [118,119], impaired osteogenic and adipogenic differentiation [120], and altered leukaemic/haematopoiesis support ability [121] highlighting their pivotal role during AML progression. Moreover, several studies have shown that BM biopsies from AML patients with increased numbers of CD271+ MSCs are associated with treatment resistance and reduced overall survival [122], with their contribution to an exacerbated production of ECM reticular fibres also being associated with therapy induction failure in these patients [123].

Indeed, accumulated evidence now shows that BMMSCs are transcriptionally [124], genetically [125], and functionally [126] altered in AML patients compared to healthy donors. Leukaemia-educated BMMSCs are misguided to actively secrete pro-inflammatory cytokines and chemokines, either as soluble factors or packed into EVs that downregulate HSCP maintenance while stimulating AML blasts homing into available haematopoietic niches. This intricate signalling network perpetuates disease by endorsing AML blasts’ outgrowth over HSCs and escape from immune control, and even sustaining LSC survival during therapy [127]. Illustrating this is increased secretion of IFN-γ triggered by the overexpression of the TWIST1 oncogene in many MDS and AML blasts [59]. Leukaemia-secreted IFN-γ seems to activate STAT1 signalling in BMMSCs, downregulating NAD(P)H quinone-oxidoreductase-1 (NQO1) redox enzymes, with a consequent increase in intracellular ROS generation [58]. Interestingly, this seems to favour BMMSCs to use OXPHOS-related proteins, strongly inhibiting their osteogenic differentiation and promoting BMMSC senescence. AML-induced BMMSC senescence seems to be a major hallmark of their re-education process. This includes the overexpression of markers related to cell cycle arrest, ROS, DNA damage, and senescence-associated secretory phenotype (SASP) [128], with heterochromatin disorganization being one of the main drivers of leukaemia-induced BMMSC senescence [129]. Indeed, Abdul-Aziz et al. [128] have described that AML-generated NOX2-derived superoxide can also trigger a pro-leukaemic p16INK4a-dependent senescence in BMMSCs. Interestingly, LSCs also seem to shed large amounts of exosomes rich in senescence-inducing proteins to further reinforce BMMSC senescence, luring them to selectively produce exosomes rich in stemness-promoting proteins to their own advantage [92]. Most importantly, targeting senescent BMMSCs directly inhibited AML blast proliferation and enhanced the survival of leukaemia-bearing mice. Taken together, this highlights the key role of a senescent environment for LSCs to thrive [128].

Concomitantly, excessive amounts of secreted IFN-γ in AML—combined with other inflammatory cytokines—leads to the polarization of the remaining BMMSCs into an anti-inflammatory secretion profile. Importantly, this seems to be able to dampen the immune response and shelter LSCs from immune control [130,131,132]. Importantly, this BMMSC secretion profile includes immunosuppressing prostaglandin E2 (PGE2), IL-6, IL-10, galectins, transforming growth factor-beta (TGF-β), tumour necrosis factor-stimulated gene-6 (TSG-6), hepatocyte growth factor (HGF), and human leukocyte antigen-G5 (HLA-G5) [60,61,62]. Additionally, BMMSCs also increase intracellular enzymes indoleamine-2,3-dioxygenase (IDO) and inducible nitric oxide synthase (iNOS) [133], as well as the production of adenosine by the ectonucleotidase CD73 [134] mediating cell–cell immune suppression.

Interestingly, many of these immunomodulatory proteins are also carried within BMMSC-shed EVs, whose packing is drastically increased, when cultured in their native hypoxic environment [103]. Indeed, the synergistic effects of combinatorial hypoxia plus inflammatory cytokine priming—similar to the microenvironment found in progressing AML—have been described to trigger BMMSC aerobic glycolysis [135] and metabolic reconfiguration [136] to fuel changes in the yield of vesicle production and lipidic profiles, driving the secretion of EVs with enhanced immunosuppressive potency [137].

Indeed, BMMSCs are master regulators of the BM haematopoietic compartment. In this regard, de Kruijf et al. demonstrated that BMMSCs control quiescent HSC cell cycle re-entry, proliferation, and mobilization from the niches, mainly through the secretion of EVs [138]. Interestingly, these authors showed that shed BMMSC-derived EVs are engulfed primarily by BM-derived macrophages—but also by endothelial cells—downregulating the expression of CXCL12, VCAM, and SCF haematopoietic niche factors in endosteal cells, and leading to HSC release and mobilization. It is interesting to note that similar mechanisms seem to be exploited by LSCs during disease progression [76,94]. Recently, Deniz et al. [72] showed that BMMSCs produce migrasomes with TSPAN4 and BMMSC markers that carry CXCL12. Interestingly, when co-cultured with KG1a leukaemic cell lines and HSPCs, these migrasomes showed the capacity to attract both cell counterparts in a CXCR4-dependent mechanism. Most importantly, leukaemic cells, but not HSPCs, were able to uptake these migrasomes, highlighting the role of distinct EV subsets in haematopoiesis but also in AML leukaemogenesis. In this line of reasoning, LSC-secreted EVs also seem to impact cell-to-cell leukaemia supportive signalling by BMMSCs at disease onset. Indeed, LSC-derived EVs have been shown to carry miR-188-5p to trigger the loss of bisecting GlcNAcylation on the MCAM (melanoma-associated cell adhesion molecule; also known as CD146) of BMMSCs by targeting the MGAT3 gene. This reshaping of the BMMSC glycocalyx seems to further enhance cell–cell membrane signalling—with MCAM on the stromal cell surface with reduced bisecting GlcNAc strongly binding to CD13+ myeloid cells—activating ERK signalling and favouring uncontrolled leukaemic blast proliferation [63].

Recently, Kargar-Sichani et al. have shown that AML cell-derived EVs also seem to modulate the BMMSC phenotype in a concentration-dependent manner [71]. These authors have observed that a lower leukaemic blast-derived EV dosage triggers BMMSC proliferation and survival through an increase in inKi-67 and BCL-2, with decreased levels of intracellular ROS. Interestingly, a higher dose of AML-EVs induces BMMSC apoptotic death via increased BAX expression. Despite both AML-EVs dosage induced BMMSCs to secrete known AML promoting cytokines, including IL-6, Gas-6, and Galectin-3, it would be interesting to determine whether this pleiotropic effect could be replicated in vivo (or in BM-mimetic ex vivo models) to ascertain whether it represents a common mechanism employed by LSCs in distinct stages of BM infiltration and disease burden.

It is important to note that distinct EV mechanisms may be more predominant in certain AML subtypes than others. In this regard, in MPN1-mutated AML, NPM1-mA was shown to recue fat mass and obesity-associated (FTO) protein from proteasomal degradation to reduce the global m^6^A abundance, leading to the activation of the PDGFRB/ERK signalling axis to autonomously drive leukaemic blast survival [139]. Most importantly, BMMSCs were also shown to reinforce this mechanism by delivering RNA m6A demethylase FTO-containing exosomes to AML blasts. Importantly, these FTO exosomes triggered m^6^A-demethylated LncRNA GLCC1, facilitating its combination with the RNA-binding protein Hu antigen R (HuR), which further reinforced the stability and de novo expression of demethylated LncRNA GLCC1 in leukaemic blasts. Consequently, demethylated LncRNA GLCC1 stabilizes the IGF2 mRNA binding protein 1 (IGF2BP1)-c-Myc complex, with downstream activation of the tumour-promoting c-Myc-associated signalling pathway translating into enhanced leukaemia aggressiveness and Ara-c chemoresistance [70].

##### Osteoprogenitors, Bone Lining Cells, and Osteoclasts

In both patients and AML mouse models, BMMSCs conspicuously exhibit a significant delay in osteogenic differentiation. Indeed, careful morphological analysis reveals a profound reshaping of endosteal BM architecture characterized by the loss of mature osteoblasts [84,140,141], concurrent accumulation of osteoprogenitors [142,143], and transiently increased osteoclastic activity [144,145].

Osteolineage cells, including committed osteoprogenitors and mature osteoblasts, produce many HSC-supporting molecules, including OPN, CXCL12, SCF, thrombopoietin (TPO), and ANGPT, controlling HSC renewal, expansion, homing, and maturation along different lineages [146,147]. Most interestingly, the stage of osteoblastic maturation seems to define their function in HSC haematopoiesis, with most immature osteoprogenitor subsets influencing HSC maintenance and proliferation [146], while mature osteoblasts appear to assist in HSCP differentiation along myeloid, erythroid, and lymphoid lineages [109,147]. Recently, Galan-Díez et al. [148] postulated that at the onset of AML, osteoblasts seem to perform a dual function: they seem to exert protective signals of an elusive nature and, at the same time, AML blasts subvert serotonin receptor signalling in mature osteoblasts through the kynurenine–HTR1B–SAA–IDO1 axis to persist within the endosteal niche. As leukaemic burden increases, the settlement of a self-perpetuating pro-inflammatory niche seems to drive mature osteoblast apoptosis, decreasing their numbers and protective signals while the kynurenine–HTR1B–SAA–IDO1 pathway is maintained (Figure 3A).

Most interestingly, Duarte et al. [84] have shown that AML blasts within endosteal regions can secrete pro-inflammatory and anti-angiogenic factors that gradually degrade the endosteal endothelium, stromal cells, and mature osteoblastic cells, whereas central marrow remains vascularized and splenic vascular niches are expanded. Most importantly, remodelled endosteal regions have a reduced capacity to support HSCs, correlating with the loss of normal haematopoiesis, with the functional rescue of these niche cells delaying AML progression.

Mechanistically, endorsing BMMSCs’ commitment to osteoblast precursors with downstream stalling of the maturation process appears to be a key step in AML progression (Figure 3B). Early on, leukaemic cells appear to disrupt bone homeostasis by secreting high amounts of CCL3 [149] and IL-1β [150] to induce the commitment of BMMSCs to osteoprogenitor cells but not mature osteoblasts [143,144,151,152]. This is further reinforced by AML cell-derived EVs carrying Bone Morphogenic Protein 2 (BMP2), a known regulator of osteogenesis and inflammation, precipitating compartmental endoplasmic reticulum stress and an unfolded protein response (UPR) in both leukaemia and BMMSC precursors [153].

Kumar et al. have shown that AML patients exhibit an increase in exosome secretion, which is associated with a reduction in osteocalcin (OCN) plasma levels [91]. These authors elegantly showed that treatment of BMMSCs with AML cell-derived exosomes decreases the expression of genes that control osteoblast maturation (OCN, Col1A1, IGF1) and support normal haematopoiesis (CXCL12, KITL, IL-7, IGF1) while increasing the expression of genes supporting AML growth (DKK1, IL-6, CCL3). Interestingly, Rab27a knockdown cancelled both the increase in SCA1+/CD146+ stromal cells and osteoblastic maturation blockage induced by DKK1 upregulation in AML, significantly extending mice survival. In support, Chetty et al. [69] have recently reported that YBX1-containing AML-sEVs are responsible for the observed osteogenic differentiation stalling of BM-MSCs. Adding to this, others have also reported that miR-4532, frequently overexpressed in AML blasts, is selectively packed and enriched in AML-secreted exosomes [64]. These AML exosomes seem to also increase the expression of DKK1 in target cells via an LDOC-dependent STAT3 signalling pathway. Interestingly, DKK is a negative regulator of Wnt signalling required for stem cell differentiation and maturation processes [65,154]. Thus, exosomal miR-4532 (and potentially others [155]) could drive the overexpression of DKK1 in pre-osteoblasts, stalling osteolineage maturation, disrupting bone mineralization, and compromising endosteal niche haematopoiesis. The importance of AML-depleted endosteal niches is thus highlighted by the delay in disease progression upon restoration of osteoblasts and/or endosteal vascularization.

Endosteal bone-degrading osteoclasts are another cell type that may play a role in AML altered bone homeostasis. These cells originate from recruited monocytes/macrophages upon inflammatory stimuli. Interestingly, leukaemic blasts seem to stimulate osteoclastogenesis in an early phase, possibly as a means to expand the central marrow cavity space (Figure 3C). Indeed, Frisch et al. observed in an AML mouse model that there is an initial and transient increase in osteoclastic cells, but as the disease progresses to overt leukaemia, osteoclastic cell numbers decline compared to healthy controls [144]. Shedding some light on the mechanisms behind this observation, Sadvakassova et al. [66] have reported that K562 erythroleukaemia cells shed exosomes carrying L-plastin, PRDX2, and PRDX4, factors that regulate osteoclastic activity [66,67,68]. Most importantly, inhibition of leukaemia exosomal release significantly decreased the osteoclastogenic capacity of K562 cells’ secretome, establishing their key role in mediating the process. Additionally, both the high levels of EPO [156,157] along with forced erythropoiesis secondary to anaemia [158] observed in AML patients can further upregulate the levels of exosomal PRDX2 shed by remaining erythroid progenitor cells to reinforce osteoclast formation and bone loss-related signalling [66,159,160].

Taken together, these data suggest that there is a deliberate shedding of EVs from leukaemic blasts, which aim to increase osteoprogenitor numbers and stall their maturation—hijacking HSC niche proliferative signalling—while targeting osteoclasts to degrade endosteal bone, increasing the available marrow space for AML outgrowth. The increased pro-leukaemia osteoprogenitors and the few reprogramed mature osteoblasts that survive inflammation fuel the signalling network required for overt AML.

#### 3.3.2. Vascular Niche Remodelling

HSCs and their LSC counterparts can be distributed along two distinct locations: the endosteal niche (in close proximity to the inner surface of the bone, rich in arterioles), and the vascular niche, located deep in the BM cavity, characterized by an extensive network of sinusoidal capillaries. At this point, it is important to specify that in both endosteal and vascular niches, HSCs/LSCs seem to reside in perivascular areas, possibly due to endothelial cell anchoring [161,162]. Proportionally to the extent of endosteal and vascular niche regions, HSCs are mainly adjacent to sinusoidal blood vessels in the BM and spleen, with a small percentage of HSCs localized near the endosteum as long-repopulating HSCs [163,164]. These HSCs are specifically associated with small arterioles in the endosteal BM, which are exclusively surrounded by rare NG2+ pericytes. Activation of the HSC cell cycle, either pharmacologically or genetically, dynamically alters their distribution from NG2+ peri-arteriolar niches to LepR+ peri-sinusoidal niches [165].

As previously pointed out by us [84], one pathognomonic alteration within AML niche remodelling is the destruction of endosteal niche vasculature along with the already stated alterations leading to endosteal bone loss over leukaemia progression. So far, milestone scRNA-seq studies that defined marrow’s cellular taxonomy of stromal compartments, BM endothelial cells (BMECs), were largely divided into two populations: Arterial Endothelial Cells (AECs) and Sinusoidal Endothelial Cells (SECs) [79,98]. In this regard, Iga et al. [162] recently extended this knowledge, further identifying 11 distinct EC subclusters by scRNA-seq, illustrating endothelial cell heterogeneity. Importantly, these authors were able to categorize them into arterial, venous, and capillary ECs, with specific attention to type-H, type-L, and type-S EC functional origin.

Noteworthy, type-H vessels, supplied by arterioles, exhibit heightened blood flow and oxygen and nutrient levels compared to type-L vessels [166,167]. These capillaries, marked by elevated expression of endomucin (Emcn) and CD31, are typically found adjacent to runt-related transcription factor 2 (Runx2)- and osterix-expressing osteoprogenitors, particularly in bone regions undergoing active growth [168]. Recently, a novel capillary subtype, termed type-S vessels, originating exclusively from the secondary ossification centre (SOC) in the epiphysis region, has also been identified [162]. Type-H and -S vessels are functionally distinct from type-L vessels, whose EC displays lower Emcn and CD31 expression, along with characteristics such as Sca-1^low^ and VEGFR3+ [165,169,170] being primarily associated with sinusoidal-like vessels [171]. Noteworthy, this illustrates well the vascular-niche heterogeneity throughout the whole BM compartment. Despite recent advances in EC subset characterization, their relation with emerging LSCs remains largely unexplored, with most studies approaching the general role of EC in AML progression instead. It would be interesting to confirm whether LSCs preferentially remodel these distinct endosteal and vascular niches to support their progression. While future studies are required to elucidate this, here, we will review the most recent data on how the distinct components of the vascular niche, including endothelial cells, perivascular pericytes, and other accessory cells such as adipocytes and neurons from the parasympathetic nerves, are re-educated by LSCs to support leukaemia in an intricate EV signalling network.

##### Endothelial Cells and Progenitors

The functional EC subsets that line the surface of distinct type-H, -S and -L blood vessels of BM control the vascular integrity. Indeed, their unique properties have capital importance for establishing the milieu for either enhanced HSC/LSC trafficking or quiescency [170]. Importantly, vessel permeability determines the exposure of adjacent niche populations such as HSCs/LSCs to plasmatic reactive oxygen species (ROS), dictating their cellular fate. In support, continuous lining arterioles (arguably associated with type-H and -S vessels) typically maintain nearby HSCs in a dormant state with minimal ROS exposure [165,172], whereas leaky sinusoids elevate (associated with type-L vessels) ROS levels in regional niche populations, prompting their proliferation and release into circulation [173] (Figure 3).

The onset of leukaemia deliberately disturbs this vascular balance. Indeed, AML patients’ BM is widely recognized by its increased microvessel density (Figure 3D), a vascular hallmark directly linked to AML prognosis in a number of studies [174,175,176]. Unfortunately, subsequent clinical trials addressing anti-angiogenic therapies observed a disappointing impact on AML patients’ survival [177,178], prompting the idea that a more complex relationship between AML and vascular niche occurs beyond simple angiogenesis. Indeed, intravital microscopy studies in AML mouse models [179], including our own [84], have shown that AML leads to a selective expansion of the vascular niche microvessel density while causing a targeted decline in endosteal niche vessels. This reduction in endosteal vessels diminishes the number of healthy HSCs, prompting AML blasts to occupy their niches for overt leukaemia. Conversely, rectifying endosteal vessel abnormalities [84] or hindering vascular-derived nitric oxide [179] rescued healthy haematopoiesis and improved the effectiveness of chemotherapy. Despite the mechanism leading to this vascular remodelling selectivity being currently unknown, emerging AML blasts were shown to directly promote angiogenesis by secreting VEGF and IL-8 soluble factors [180,181] or EVs enriched in several miRs, mRNA VEGF, VEFGR, and angiopoietins [24,25,77]. In this regard, Umetsu et al. [77] reported that the K562 leukaemic cell line shed CD63+ EVs carrying miR-92a that, upon internalization by HUVECs, enhance endothelial cell migration and tube formation, but not their proliferation, illustrating the dysregulated angiogenic signalling perpetuated by leukaemia. Along the same line, Li et al. [78] have recently reported that AML cell-derived EVs can also trigger a strong angiogenic response from HUVECs in an in vitro setting. Interestingly, the release of pro-angiogenic EVs seems to be controlled by p62 expression in AML blasts. Concomitantly, these authors observed by bioinformatic analysis that these AML cell-derived EVs carried excessive amounts of hsa-miR-3064-3p, hsa-miR-339-5p, and hsa-miR-3622a-5p, implying that the MAPK signalling pathway could play a significant role in the observed angiogenic response. Nevertheless, the direct relationship between these specific vesicular miRNAs and angiogenesis in AML still remains to be experimentally demonstrated.

Remarkably, several lines of evidence now illustrate the intimate relationship between LSCs, their HSC counterparts, and EC cells, probably owing to their shared ontogeny [170,182]. Not surprisingly, leukaemic blasts express several VEGF family members and their cognate receptors [183], with elevated levels of VEGF-A and VEGF-C in leukaemic blasts being associated with a worse patient outcome [184]. Importantly, leukaemic cells also express most adhesion molecules mediating physical interaction with ECs (e.g., VLA-4/VCAM-1; CD44/E-selectin; etc., reviewed in [185,186]), with blasts located in close proximity to ECs being typically chemoresistant. Indeed, perivascular and endosteal stroma become progressively abnormal with increasing blast burden, as previously shown by us [84]. In this late stage, there is an inefficient formation/retraction of vessel sprouts evident in highly infiltrated areas, leading to detachment of ECs and vascular collapse. Interestingly, upon vessel collapse, abundant 1 to 4 μm sized cellular debris of endothelial origin was observed in the vascular lumen of AML-burdened mice. There is a high likelihood that these particles were endothelial cell-derived EVs as they maintained expression of CD31/endomucin phenotypic endothelial markers and contained nucleic acids within an intact membrane. Nevertheless, their role in AML-remodelled niches remains unknown.

Along this line of reasoning, Huang et al. [75] importantly showed in an MLL-AF9-induced AML mouse model that blocking sEV secretion from ECs, but not perivascular cells, megakaryocytes, or spleen stromal cells, markedly delayed leukaemia progression. Indeed, these authors have elegantly shown that AML-educated endothelial cells release high levels of angiopoietin-like 2 (ANGPTL2)-containing small EVs—a process governed by Vps33b exosomal protein machinery. In return, EC-secreted ANGPTL2-sEVs interact with the surface of LSC leukocyte immunoglobulin-like receptor B2 (LILRB2) to drive disease progression. Noteworthy, this vascular niche reprograming seems to specifically target ECs to express much higher levels of exosomal Vps33b proteins and Angptl2 in leukaemic BM compared to healthy controls. Importantly, this strongly suggests that leukaemic blasts require ANGPTL2-sEVs to survive, and that they therefore stimulate ECs to shed exacerbated amounts of ANGPTL2-sEVs, possibly explaining why AML selectively enhances vascular niche microvessel density. Moreover, these authors showed that the expression of LILRB3 was higher in AML blasts than in normal HSC counterparts and myeloid progenitors, which could explain why ANGPTL2-sEVs impacted leukaemic blasts but not normal haematopoiesis.

In support, Fang et al. [24] also demonstrated that exosomes derived from Acute Promyelocytic Leukaemia (APL) NB4 cells enhance the angiogenic capacity of ECs and thus prolong the survival of APL cells in vitro. Most importantly, these authors showed that, in a steady state, NB4 cell-derived EVs have 17 angiogenic transcripts upregulated in comparison with their parental cells. Interestingly, the secretion profile of EVs changed upon ATRA treatment, with 42 angiogenic transcripts being upregulated in NB4-EVs compared to the corresponding APL cells. This evidence clearly highlights the dynamic nature of vascular remodelling and the importance of EV networks in perpetuating LSC survival during therapy. This endothelial–haematopoietic relationship further deepens with reports demonstrating that AML blasts are themselves able to integrate into the BM vasculature, becoming vascular tissue-associated AML cells [187,188]. In this extraordinary process, transdifferentiated/vessel-fused AML cells acquire several endothelial cell-like characteristics—including the upregulation of CD105—adopting a quiescent phenotype. Most importantly, these EC-fused AML cells retain the ability to trigger a full-blown relapse upon transplantation, illustrating their potential to act as a hidden reservoir of residual disease. Interestingly, Xu et al. [189] have recently shown in an MLL-AF9 AML mouse model that nearly half of AML blasts in resting phase were observed within 0 to 4 μm from blood vessels, suggesting that quiescent leukaemic cells make cell–cell contact with ECs to evade therapy. Indeed, several mRNAs associated with the development of leukaemia, including Dusp6, Klf4, and Plxnb2, were significantly elevated in ECs and resting leukaemia cells after chemotherapy. Most interestingly, upon treatment, quiescent AML cells preferentially migrated to the endosteal region (34.8% in the endosteal vs. 6.6% in the BM cavity), whereas cycling AML cells were primarily concentrated in the centre of the BM cavity (4.9% in the endosteal area vs. 36.5% in the BM cavity). Interestingly, the exact signalling pathways employed by residual/quiescent LSCs to relapse seem to be highly AML-subtype-specific. In this regard, Kellaway et al. have recently shown that t(8;21) AML relapse relies on the specific interplay of the driver mutation with the stem cell program being triggered by aberrant activation of VEGF and IL-5 signalling, most probably provided from the vascular niche [190].

Beyond the endothelial–haematopoietic axis, AML blasts also seem to be able to re-educate nearby BMMSCs to secrete pro-angiogenic EVs—reinforcing the pro-angiogenic loop—driving EC glycolysis, proliferation, and network formation [25,78]. In this regard, Huan et al. [76] discovered that AML cell-derived exosomes carry mRNA encoding the insulin-like growth factor-1 receptor (IGF-1R) that, upon internalization by BMMSCs, lead to both increased proliferation and secretion of VEGF to further reinforce local angiogenesis.

##### Adipocytes

BM adipocytes arise through the differentiation of a subset of leptin receptor-positive (LepR+) BMMSCs [191]. Indeed, several studies have reported that while peri-arteriolar BMMSCs display a propensity to undergo osteogenesis, sinusoidal BMMSCs show an enhanced adipogenic profile [79,98,192]. As individuals age, the proportion of adipose tissue within distinct parts of the BM—red versus yellow marrow—undergoes significant changes, with an increased preponderance of the latter. Adipocytes are less frequent in red marrow, where haematopoiesis and bone remodelling are more active, accounting for up to 45% of the marrow space. In contrast, yellow marrow, characterized by minimal haematopoietic activity, is mostly composed of densely packed adipocytes, filling up to 90% of the marrow compartment [193,194]. Despite their high proportion in the marrow space, not much attention has been paid to the role of BM adipocytes so far. However, recent reports seem to indicate that these cells may play a pivotal role in the BM vascular niche under stressful conditions, secreting pro-inflammatory cytokines [195] and EVs [196] and influencing osteogenesis, haematopoiesis, and the progression of AML [124].

BM adipocytes secrete fatty acids, adipokines, cytokines, and EVs, with a great influence over the distinct BM niches [reviewed in [197,198]]. Although the role of BM adipocytes in regulating/inhibiting haematopoiesis is still controversial [199,200], they seem to be able to boost haematopoiesis under stressful conditions through the secretion of SCF at similar levels to their BMMSC precursors [191]. In support, Tikhonova et al. [79] have also observed a transcriptional remodelling of the BM niche components with adipocytic skewing of the perivascular cells. Interestingly, this Lepr^+^ BMMSC adipocytic skewing seems to prematurely induce a myeloid transcriptional program of surrounding HSCs. Importantly, using scRNA-seq, they identified two adipocytic-primed clusters of Lepr^+^ BMMSCs as a major source of pro-haematopoietic factors, including Cxcl12, SCF, IL-7, IL-15, M-CSF, BMP-4, and CCL-2 [79].

AML blasts seem to exploit this adipocyte feature to their advantage (Figure 3E). Indeed, Lu et al. [201] observed that the proportion of small BM adipocytes in AML patients is significantly higher compared to healthy BM controls. Most importantly, AML patients displaying an increased amount of small BM adipocytes correlated with a shorter overall survival and relapse-free survival, with patients who achieve complete remission displaying a diminished amount of these adipocytes compared to therapy-refractory individuals. These observations pinpoint a possible role of small adipocytes in mediating early AML progression and therapy resistance. Mechanistically, AML blasts seem to reprogram the BM adipocytes to secrete adiponectin and other inflammatory cytokines that inhibit normal haematopoiesis [202,203]. Simultaneously, AML blasts secrete high levels of GDF15 to trigger the morphological remodelling of large marrow adipocytes into smaller ones [80]. This may be an initiating factor to signal the breakdown of triglycerides stored in BM adipocytes, leading to lipolysis with a subsequent release of fatty acids (FAs) into the vascular microenvironment [203]. The transportation of these FAs out of adipocytes is then facilitated by the chaperone protein FABP4, with its upregulation in blasts near adipocytes. This upregulated FABP4 in AML blasts is then exploited to transport adipocyte-derived FAs to the mitochondria within leukaemic cells, where blast mitochondria employ FAs as a substrate for β-oxidation, generating the energy needed for overt leukaemia growth [203]. In return, interaction with adipocytes triggers AML blast upregulation of PPARγ, CD36, and BCL2 proteins, required for resisting apoptotic stimuli and supporting unrestrained disease progression [204]. Interestingly, Clement et al. [205] have recently shown that adipocytes shed EVs that can stimulate fatty acid oxidation in cancer cells—improving their motility and aggressiveness—by providing both enzymes and substrates.

Within this framework, AML blast-derived EVs have recently been shown to further reinforce this adipogenic pathway through active regulation of ancestral BMMSC differentiation. In support, Zhang et al. [115] have shown that both BMMSCs isolated from AML patients and BMMSCs isolated from healthy donors co-cultured with AML cells exhibit an increased ability to differentiate into adipocytes in detriment to osteoblastic differentiation. Interestingly, AML cell-derived exosomes triggered healthy BMMSCs to upregulate the expression of genes that favours adipogenic differentiation and leukaemia engraftment, including IL6, GDF15, CEBPα, PPARγ, COL10, MMP8, and ACO2, with downregulation of osteoblastic/HSC-supportive genes such as SDF1α, BMP4, WNT5A, and LDHA2 [115]. Importantly, these instrumental phenotypic changes in AML-educated MSCs were significantly diminished upon silencing exosomal Rab27a proteins in AML blasts in vitro while prolonging the survival of Hoxa9/Meis1-driven AML mice, highlighting the key role of the leukaemic EV network in driving this process [115].

Recently, Gangadaran et al. [81] demonstrated that adipose tissue-derived stem cells shed EVs that can also deliver angiogenic proteins such as IL-8, CCL2, TIMP-1, TIMP-2, and VEGF-D to nearby endothelial cells enhancing their maturation and tube-like formation, promoting angiogenesis both in vitro and in vivo. Despite this proof of concept, it would be important to confirm whether this mechanism is also exploited in a leukaemia environment.

##### Sympathetic Neurons

The BM is highly innervated by sympathetic nerve fibres, which infiltrate the BM through the nutrient foramen. Together with NG2^+^ pericytes, sympathetic neurons are located around the arterioles within the BM niche [206]. This spatial organization allows for precise regulation of HSC activity and mobilization in response to physiological cues. Sympathetic nerve fibres release norepinephrine (NE), which facilitates HSCP migration from the BM towards extramedullary sites [207]. The binding of NE to the β3-adrenergic receptor, expressed by BMMSCs, results in a downregulation of CXCL12 expression and promotes HSC mobility and proliferation.

AML disrupts normal haematopoiesis by inducing neuropathy in the BM, altering sympathetic innervation and contributing to disease progression (Figure 3F). Hanoun et al. [208] have shown that AML reduces the density of sympathetic nerve fibres in BM niches. Upon depletion of niche cells responsible for maintaining healthy HSCs, the progression of AML is reinforced. The role of EVs in the crosstalk between the peripheral nervous system (PNS) and cancer cells has been recently highlighted in other types of cancer [209]. Cancer cell-derived EVs have been shown to induce neuritogenesis by delivering miRNAs [210] or axon guidance proteins [211], facilitating tumorigenesis, tumour-associated pain, and chemotherapy resistance [211,212]. Recently, in a preliminary observation, Egyed et al. have described a high density of atypical small EVs in the cerebrospinal fluid of childhood acute lymphoblastic leukaemia patients that had refractory central nervous system involvement compared with the ones that did not [213]. Although sympathetic neurons have been recently described to release EVs [214], their impact on AML progression remains unexplored.

## 4. Emerging Organ-on-a-Chip Technologies to Unravel EV Signalling Networks

While mouse models have shed light on AML’s niche dependencies, they fall short of accurately mirroring the human BM microenvironment due to inherent biological differences [215]. Conversely, longitudinal monitoring of AML niche dynamics in patients is hindered by ethical, logistical, and technical challenges, including the invasiveness of repeated biopsies and the complexity of tracking detailed cell interactions over time.

Humanized organ-on-chip (OoC) bone marrow models are emerging as a powerful tool for conducting innovative studies focused on the AML niche. OoC technology mimics organ structures by incorporating cells and tissue components into a device which can model the structure, environment, and function of the organ with unprecedented control over culture conditions. BM OoC approaches typically integrate multiple cell types within tissue engineered constructs that provide the structure, arrangement, and biomechanical forces resembling the native BM [216]. This often involves using microfluidic technology to mimic the shear stress induced by the capillary blood or interstitial flow sensed in their native environments [217]. Indeed, being able to replicate both the architecture of the tissue and the biomechanical forces to which it is subjected sets OoCs apart from two- and even three-dimensional cell culture, since cell function, cell–cell communication, and, ultimately, the whole haematopoietic process are heavily impacted by their spatial and biomechanical environment [218].

In support, healthy human BM has been modelled in OoC devices by incorporating HPSCs, MSCs, osteoblasts, and endothelial cells in scaffolds or matrices with a similar architecture to the native tissue [219]. Some approaches model the individual haematopoietic niches [220], while others incorporate both the endosteal and perivascular niches [217]. BM OoC models have also shown the capacity to replicate BM function, particularly in maintaining and inducing the differentiation of HSCs and HPSCs, and to respond to certain signals in the same way as the human BM niche [217]. Since the BM’s extracellular matrix (ECM) is crucial in maintaining tissue shape and mechanical integrity and in regulating cellular behaviour, recapitulating its properties is essential for an accurate modelling of BM niches. Hydrogels from natural polymers, such as fibrin [220,221], alginate [222], and type I collagen [223,224], have successfully been used in these advanced models to mimic native ECM.

In leukaemia modelling, Ma et al. [225] have developed an OoC device to recreate the environment of the BM niche in B-cell acute lymphoblastic leukaemia (B-ALL). Importantly, they were able to successfully model the BM niche’s components, showing heterogeneity in niche properties depending on the leukaemic B-cell lines used. Furthermore, the authors were able to accurately map the differences between the distinct engineered niches. Given the critical relationship between BM niche properties and leukaemic cell survival, OoC tools could be used for greater understanding of chemotherapy resistance in B-ALL and other types of leukaemia such as AML. Combining sophisticated OoC models with advanced EV isolation methods [226,227,228] and single-EV analysis tools [229,230,231,232,233,234,235] could pave the way for the establishment of innovative platforms to unravel the mechanisms of EV-mediated drug resistance intricacies, LSC dormancy, and relapse in human AML [225].

## 5. Conclusions and Perspectives

Accumulated evidence clearly demonstrates the imperative need of leukaemic blasts to orchestrate a plethora of reprogramming events—through extracellular vesicles—at distinct haematopoietic niches to sustain, proliferate, and progress towards overt disease. While pre-clinical models using mice engrafted with human AML cells (xenograft models) highlighted the crucial impact of the EV-mediated leukaemic niche’s establishment on disease development, a comprehensive understanding of human-specific niche signalling networks operating in distinct AML subtypes remains elusive. It is reasonable to anticipate that bridging this critical knowledge gap will hold great promise for revolutionizing the way we approach AML clinical treatment in the coming years.

While we have outlined a comprehensive roadmap of the currently known EV-mediated mechanisms governing AML cell–stroma interactions, it is important to acknowledge that the complete picture is undoubtedly far more intricate. Our exploration of AML niche-mediated signalling is merely scratching the surface of this complexity. However, the observation that leukaemic cell-derived EV infusion in mouse models is capable of inducing pathognomonic alterations within the BM—comparable to those triggered by LSCs alone—underscores its significance in driving AML progression, therapy resistance, and clinical relapse. This significantly emphasizes the importance of continued research in EV signalling mechanisms for the AML field.

Within this framework, we know that AML blasts shed many types of EVs that operate on multiple fronts with apparently contrasting effects. Indeed, LSCs release exosomes carrying stemness factors that govern their own perpetuation, influence endothelial cells and pro-angiogenic behaviour, yet they also use EVs to dispose tumour suppressor elements, triggering cell senescence and the SASP secretion profile. Additional efforts should be made in deciphering on how AML blasts distinctly regulate the multiple endothelial cell subsets that originate type-H, -S, and -L blood vessels. Are they distinctly affected? How is EV signalling altered, and to what end? Many of these questions remain largely unexplored, as we still do not comprehend how and why the endosteal blood vessels, but not the vascular niche vasculature, are systematically crippled during AML progression. Additionally, it will be important to gain a deeper understanding of the role of BM innervation, regulating bone turnover and HSC dynamics, alongside a more thorough comprehension of the contribution of adipocytes—one of the most abundant cell types in the BM—in fuelling AML progression through fatty acid metabolism. Addressing this could catalyse the development of promising niche cell-targeting therapies. Adding “insult to injury”, recent studies [82] come into place questioning the passive role of niche-dislodged HSCs during AML progression. This adds another layer of complexity on the contribution of the remaining “healthy HSCs” to residual disease perpetuation and post-treatment relapse. Clearly, the specific details of many niche EV reprograming pathways employed by AML, including the types of EVs involved and the cargo they carry, still remain blurred at best.

To this regard, the limitations of animal models in recapitulating the complexities of human AML seem to require a shift towards advanced humanized OoC BM models. Xenograft models, while informative, often lack the intricate interplay between human AML cells and the human BM microenvironment and hamper the detailed analysis of the leukaemic microenvironment. Within this context, humanized OoC models can offer a more ethical and scientifically relevant environment to explore altered niche signalling networks and test novel therapeutic interventions. Additionally, organ-on-chip integration with powerful single-EV analysis technologies could allow for a more personalized approach by incorporating patient-derived AML cells and stromal cells, enabling the evaluation of targeted therapies in a controlled setting that closely mimics the individual patient’s disease. Indeed, levering these new technologies will enable us to pinpoint the specific cell types that produce the EVs that are most crucial for LSC survival.

Importantly, we can further exploit this knowledge to develop novel therapeutic approaches by engineering these naturally AML cell-targeting EVs to act as Trojan horses, delivering engineered biologics like siRNAs (small interfering RNAs) that silence key survival genes in LSCs or chemotherapeutic drugs encapsulated within the EV membrane. This targeted approach could eliminate quiescent and proliferative LSCs with minimal off-target effects on healthy cells. Alternatively, pharmacological approaches could also be explored to silence specific EV signalling pathways during induction and consolidation therapy. For instance, drugs that target Rab GTPases, essential for EV biogenesis, or specific ESCRT complex proteins (involved in sorting cargo into EVs) could be used to disrupt AML blast-derived EV production and cripple their communication with the niche. The potential off-target effect of such intervention should be carefully weighted as it impacts other key processes in homeostasis. As we continue to close the knowledge gap in this field, these EV-based therapies could emerge as powerful adjuvants, significantly enhancing chemotherapy efficacy and offering new avenues for improving treatment outcomes.

Noteworthy, building on these insights from EV niche signalling networks could go beyond the improvement of conventional chemotherapy. For instance, pre-conditioning the BM niche through “EV-based niche regeneration” prior to allogeneic bone marrow transplantation (allo-BMT) could also be used to improve engraftment success, decrease post-transplant relapses, and improve patients’ outcomes.

Taken together, deciphering the intricate communication network within the leukaemic niche offers a new frontier for tackling AML. By harnessing the power of advanced technologies, liquid biopsies, and innovative therapeutic strategies, we strive to develop new approaches to eradicate LSCs and significantly improve patient survival. This holistic approach promises a new era of personalized and niche-targeted therapies for AML, offering hope for eradicating leukaemia.
